# *Citrus clementine* Peel Essential Oil Ameliorates Potassium Dichromate-Induced Lung Injury: Insights into the PI3K/AKT Pathway

**DOI:** 10.3390/metabo14010068

**Published:** 2024-01-19

**Authors:** Hany G. Attia, Suzan M. El-Morshedy, Ahmed M. Nagy, Ammar M. Ibrahim, Mohamed Aleraky, Sahar S. Abdelrahman, Samir M. Osman, Saeed M. Alasmari, Mohamed A. El Raey, Mohamed F. Abdelhameed

**Affiliations:** 1 Department of Pharmacognosy, College of Pharmacy, Najran University, Najran 1988, Saudi Arabia; 2 Clinical Pathology Department, National Liver Institute, Menoufia University, Menoufia 32511, Egypt; suzan.elmorshedy75@yahoo.com; 3 Department of Animal Reproduction & AI, Veterinary Research Institute, National Research Center, 33 El Bohouth St., Dokki, Cairo 12622, Egypt; an.nagy@nrc.sci.eg; 4 Applied Medical Sciences College, Najran University, Najran 55461, Saudi Arabia; mfarrag@nu.edu.sa (A.M.I.); smalasmari@nu.edu.sa (S.M.A.); 5 Department of Clinical Pathology, Al-Azhar University, New Damietta 11651, Egypt; maleraky@nu.edu.sa; 6 Department of Pathology, Faculty of Veterinary Medicine, Cairo University, Giza 12613, Egypt; saharsamirmah1@cu.edu.eg; 7 Department of Pharmacognosy, Faculty of Pharmacy, Oct. 6 University, Giza 12585, Egypt; samirothman1965@yahoo.co.uk; 8 Department of Phytochemistry and Plant Systematics, Pharmaceutical Division, National Research Centre, Dokki, Cairo 12622, Egypt; 9 Pharmacology Department, National Research Centre, 33 El Bohouth St., Dokki, Cairo 12622, Egypt; mf.abdelhameed@nrc.sci.eg

**Keywords:** lung injury, potassium dichromate, AKT, PI3K, oxidative stress, *Citrus clementine*

## Abstract

Acute Lung Injury (ALI) is a life-threatening syndrome that has been identified as a potential complication of COVID-19. There is a critical need to shed light on the underlying mechanistic pathways and explore novel therapeutic strategies. This study aimed to examine the potential therapeutic effects of *Citrus clementine* essential oil (CCEO) in treating potassium dichromate (PDC)-induced ALI. The chemical profile of CCEO was created through GC–MS analysis. An in vivo study in rats was conducted to evaluate the effect of CCEO administrated via two different delivery systems (oral/inhalation) in mitigating acute lung injury (ALI) induced by intranasal instillation of PDC. Eight volatile compounds were identified, with monoterpene hydrocarbons accounting for 97.03% of the identified constituents, including 88.84% of D-limonene. CCEO at doses of 100 and 200 mg/kg bw exhibited antioxidant and anti-inflammatory properties. These significant antioxidant properties were revealed through the reduction of malondialdehyde (MDA) and the restoration of reduced glutathione (GSH). In addition, inflammation reduction was observed by decreasing levels of cytokines tumor necrosis factor-α and tumor growth factor-β (TNF-α and TGF-β), along with an increase in phosphatidylinositide-3-kinase (PI3K) and Akt overexpression in lung tissue homogenate, in both oral and inhalation routes, compared to the PDC-induced group. These results were supported by histopathological studies and immunohistochemical assessment of TGF-β levels in lung tissues. These findings revealed that CCEO plays an integral role in relieving ALI induced by intranasal PDC and suggests it as a promising remedy.

## 1. Introduction

Acute Lung Injury (ALI) is a life-threatening syndrome characterized by diffuse pulmonary edema, hypoxemia, and respiratory insufficiency, which in some cases can progress to acute respiratory distress syndrome (ARDS) [1]. ALI is considered the most severe form of viral infection, including COVID-19 [2]. Currently, there is no specific treatment for ALI due to the diverse causes and complex manifestations. Supportive treatment, such as mechanical ventilation and the use of corticosteroids, remains the current approach for managing ALI [3]. However, the high morbidity and mortality rates of ALI remain significant concerns, prompting critical attention to investigate ALI pathways and explore novel lines of treatment.

The pathogenesis of ALI remains incompletely understood. Oxidative stress is considered the main underlying mechanism of all destructive alignments that lead to inflammatory conditions including ALI. Among different oxidizing agents, potassium dichromate is a highly toxic compound that generates reactive oxygen species (ROS) and causes cellular damage. The widespread environmental distribution of chromium, especially in the form of PDC used in various industrial applications, has increased interest in understanding its toxicity and biological effects [4]. Inhalation or exposure to PDC can result in severe ALI characterized by inflammation, oxidative stress, and damage to lung tissues. Understanding the molecular mechanisms involved in PDC-induced lung injury is crucial for identifying potential therapeutic targets [5].

The resultant ROS can damage the alveolar epithelium and vascular endothelial cells, leading to pulmonary edema [6]. Additionally, ROS can stimulate the expression of pro-inflammatory cytokines, thereby promoting the inflammatory cascade. The inflammatory process triggered by cytokines and pro-inflammatory factors, including tumor necrosis factor (TNF)-α, interleukin (IL)-6, tumor growth factor (TGF)-β, and nuclear factor kappa B (NF-κB), plays a crucial role in the pathway of ALI. Moreover, previous research has demonstrated the involvement of the phosphatidylinositide-3-kinase (PI3K)/AKT signaling pathway in ALI, with inhibition of this pathway showing protective effects against acute inflammatory responses. The use of antioxidants plays a crucial role in mitigating the detrimental effects of ALI on pulmonary vascular endothelial cells and restoring the balance with oxidative stress factors. In recent years, there has been growing interest in the utilization of natural medicines for the treatment of various diseases, including ALI [7].

*Citrus clementine* belongs to the Rutaceae family and has a high content of essential oils and aromatic compounds, it exhibits robust antioxidant, antimicrobial, analgesic, neuroprotective, and antiviral effects [8].

The delivery system plays a crucial role in the efficacy and minimization of side effects of medications. In the treatment of respiratory problems, the oral and inhalation routes are the most commonly prescribed. However, the preferred method of administering treatment for lung diseases, such as asthma, chronic obstructive pulmonary disease (COPD), and ALI, is through pulmonary delivery. This direct delivery of medication to the lungs offers several advantages, including the faster onset of action and greater effectiveness at lower doses, thereby minimizing potential adverse effects on the rest of the body [9,10]. Therefore, this study aims to provide new insights into the anti-inflammatory effect of limonene-rich essential oils extracted from *C. clementine* using two distinct delivery systems, oral and inhalation routes, in PDC-induced ALI, focusing on the PI3K/AKT signaling pathway in rats.

## 2. Materials and Methods

### 2.1. Chemicals and Kits

PDC was purchased from Sigma Aldrich Chemical Co. (Burlington, MA, USA). The lipid peroxidation marker (MDA) and reduced glutathione (GSH) were obtained from Biodiagnostic (Cairo, Egypt). TNF-α and TGF-β were purchased from specific SunRed ELISA kits, Shanghai, China. All experiments were performed according to the manufacturer’s instructions.

### 2.2. Plant Materials

Ripe fruits of *Citrus clementine* Hort. ex Tan. were collected from a private farm in Ghoreib village, Zifta, Gharbia governorate, Egypt, at the end of December 2022. The plant material was identified by Prof. Dr. Sayed Hassan, Professor of Pomology, Pomology Department, Agricultural and Biology Institute, National Research Centre, Dokki, Cairo, Egypt. The peel was separated from the flesh manually.

### 2.3. Animals

Female Wister albino rats weighing between 140 and 150 g were sourced from the Animal House of the National Research Centre in Cairo, Egypt. The rats were kept in groups, maintained under controlled conditions of temperature (24 ± 2 °C) and lighting (12 h light/dark cycle), and provided with unrestricted access to standard laboratory rodent food and water. All studies were conducted in accordance with ethical guidelines for the care and use of experimental animals approved by the Research Ethics Committee [Approval No: PRE-Ph-2312016].

### 2.4. Extraction

Fresh *Citrus clementine* peels weighing 1.5 kg were mixed in a blender with 7.5 L of 95% ethanol. The mixture was then filtered, and 0.5 L of H_2_O was added to the filtrate. The resulting filtrate appeared turbid and was further partitioned with petroleum ether (40–60) using the method described by [11]. The petroleum ether fraction was evaporated under vacuum using a rotary evaporator at 30 °C, resulting in 30 mL of *Citrus clementine* peels essential oil (CCEO).

### 2.5. Gas Chromatography–Mass Spectrometry Analysis (GC/MS)

A gas chromatograph (Agilent 8890 GC System) coupled with a mass spectrometer (Agilent 5977B GC/MSD) was utilized in the study. The system was equipped with an HP5MS fused silica capillary column (30 m × 0.25 mm × 0.25 µm), Agilent Technology, Santa Clara, CA, USA. The oven temperature was initially set at 50 °C and then programmed to increase from 50 °C to 200 °C at a rate of 5 °C/min. Subsequently, it was further increased from 200 °C to 280 °C at a rate of 10 °C/min and held at 280 °C for 7 min. Helium was employed as the carrier gas with a flow rate of 1.0 mL/min. The essential oil was dissolved in diethyl ether, with a concentration of 20 µL essential oil per milliliter of diethyl ether. Then, 1 µL of this solution was injected into the GC with a split ratio of 1:50. The injection temperature was set at 230 °C. Mass spectra were acquired in the electron impact mode (EI) at 70 eV, scanning the mass-to-charge ratio (*m*/*z*) range from 39 to 500 amu. The peaks in the chromatogram were identified by comparing them with data from the library of mass spectra provided by the National Institute of Standards and Technology (NIST). The Kovats retention indices (RIs) were experimentally measured and compared to the values reported in the literature, using C8-C30 n-alkanes as a reference [12].

### 2.6. Experimental Design

Forty-eight rats were randomly allocated into six groups (n = 8). The 1st group received intranasal (i.n.) saline and served as the normal group, while the 2nd group received PDC intranasally as a single dose of 2 mg/kg and served as the acute lung injury model group [5]. The 3rd–4th groups were administered CCEO at doses of 100 and 200 mg/kg bw; orally), respectively, for 7 days before PDC instillation while the 5th–6th groups were administered CCEO at doses of 100 and 200 mg/kg bw by inhalation, respectively, for 7 days before PDC instillation.

### 2.7. Biochemical Determination

Rats were sacrificed by decapitation under light anesthesia. The lungs were washed with saline and placed in ice-cold phosphate buffer (pH 7.4) to prepare 20% homogenate using a homogenizer (Heidolph, DIAX 900, Burladingen, Germany). The homogenates were centrifuged at 2000× *g* for 20 min at 4 °C. The supernatant was collected and stored at −80 °C [13] and then used for estimation of lung contents of TNF-α and TGF-β using NOVA (Beijing, China) ELISA kits and of IP3k using SunRed Biotechnology Company ELISA kits. Another part of lung tissue was kept in 10% formalin-saline for histopathological examination.

### 2.8. Real-Time Polymerase Chain Reaction (PCR) Quantification of Protein Kinase B (Akt)

The mRNA expression level of the Akt gene was evaluated using real-time PCR, and the results were normalized using the housekeeping gene GAPDH as an internal control. RNA containing Akt transcripts was extracted from brain tissue using Trizol reagent. The RNA was then reverse-transcribed using M-MLV reverse transcriptase (Invitrogen, Carlsbad, CA, USA), and the resulting cDNA was used for PCR amplification with specific primers targeting the Akt gene. The quantification of Akt gene expression was performed using the Akt RT-PCR fluorescence diagnostic kit (Cat. No. A.B 517302) according to the manufacturer’s instructions. The PCR amplification consisted of 40 cycles, with each cycle consisting of denaturation at 95 °C for 5 s, annealing at 61 °C for 1 s, and extension at 72 °C for 30 s. Following the amplification cycles, a final extension step was performed at 60 °C for 1 min and 72 °C for 10 min. The specific primers used for amplification were as follows: forward primer 5′-GTGGCAAGATGTGTATGAG-3′ and reverse primer 5′-CTGGCTGAGTAGGAGAAC-3′. The amplification and detection of PCR products were carried out using the Rotor-Gene Q5 plex real-time Rotary analyzer (Corbett Life Sciences, San Francisco, CA, USA).

### 2.9. Histopathological and Immunohistochemical Evaluations

Different lobes of lungs of five animals from each group were fixed in 10% neutral buffered formalin, then regularly processed for paraffin blocks. The later blocks were sectioned at 4–5 μm thick, and they were stained with haematoxylin and eosin (H&E) for histopathological examination [14]. The obtained slides were examined blindly without identity using a binocular Olympus CX31 microscope. A semi-quantitative lesion-score system ranging from 0 to 4 was employed to evaluate the histopathological changes observed, following a method described by [15] with some modifications. In this scoring system, a score of 0 indicated normal tissue structure, 1 represented minimal alterations with sporadic lesions in limited areas, 2 indicated slight alterations with evident changes in limited areas, 3 denoted moderate alterations with lesions confined to one-third of the total lobe area, and 4 indicated marked alterations with extensive lesions covering more than one-third of the lobe.

For immunohistochemical analysis, paraffin sections were subjected to antigen retrieval by heating in a microwave oven at 720 W for 25 min. Subsequently, the sections were incubated overnight at 4 °C with a rabbit monoclonal antibody specific to TGF-β1 (Abcam, Cambridge, MA, USA, ab184787) at a dilution of 1:200. Following PBS washing, the sections were incubated with the corresponding biotinylated secondary antibody (Dako Corp., Nowy Sącz, Poland) at a dilution of 1:200 and a streptavidin/ALP (alkaline phosphatase) complex (Dako Corp.) at a dilution of 1:200 at room temperature for 30 min. The binding sites of the antibody were visualized using DAB (Sigma) and counterstained with hematoxylin for 2–3 min. Subsequently, the samples were dehydrated using increasing concentrations of ethanol, immersed twice in xylene for 5 min at room temperature, mounted, and examined under a light microscope (Olympus BX50, Tokyo, Japan) [16]. The extent of marker expression was quantified by measuring the optical density (OD) of the positive brown area in seven high-power microscopic fields using image analysis software (Image J, 1.46a, NIH, Bethesda, MD, USA).

### 2.10. Statistical Analysis

All experiments were performed in triplicates and the results were presented as mean ± SD. The statistical significance of differences was calculated and analyzed by one-way analysis of variance (ANOVA), followed by Tukey’s multiple comparisons test using GraphPad Prism 9 (GraphPad Software, Inc., San Diego, CA, USA). Values of *p* < 0.05 were considered to be significant. While the nonparametric data were analyzed and compared using the Kruskal–Wallis H test followed by the Mann–Whitney U test and represented as median ± IQR (interquartile range) as well as median ± range.

## 3. Results

### 3.1. Chemical Profile of CCEO

**Figure 1:**
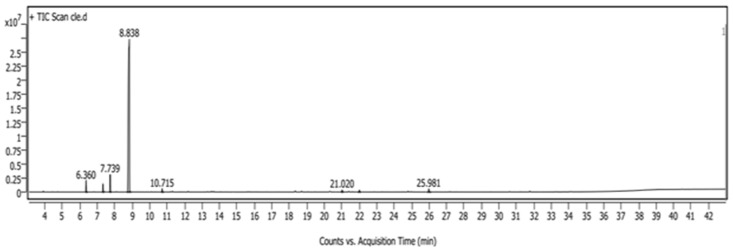
GC/MS chromatogram of *Citrus clementine* essential oils.

The essential oils of CCEO were analyzed by GC/MS (Figure 1). Eight volatile compounds were identified, as shown in Table 1.

The largest major class of the identified essential oils was monoterpene hydrocarbons, representing more than 97.03% of the volatile oils’ identified content. D-limonene was the most abundant compound at more than 88%. Other minor classes such as oxygenated monoterpenes were detected. Other monoterpenes identified were *β*-myrcene, the second most abundant compound (3.94%), followed by α-pinene (2.48%) and sabinene (1.77%), along with oxygenated monoterpene linalool which was less than 1% (0.86%). In addition to other minor products, each compound was less than 1% including sesquiterpenes (*β*-copaene and *β*-cadinene), oxygenated sesquiterpenes, and aldehyde (2,6-dimethyl-10-methylene-2,6,11-dodecatrienal; *β*-sinensal).

### 3.2. Biological Results

Swiss albino mice were administered gradient oral doses of CCEO, up to 2000 mg/kg bw, without any observable signs of toxicity or mortality within 24 h. From these observations, it can be concluded that the median lethal dose (LD50) of CCEO in mice is expected to be higher than 2000 mg/kg. According to classification criteria, as reported in previous literature, substances with LD50 values exceeding 50 mg/kg body weight are generally considered to be non-toxic [13,17].

#### 3.2.1. LD_50_ Result

##### Assessment of MDA and GSH Lung Contents 

**Figure 2:**
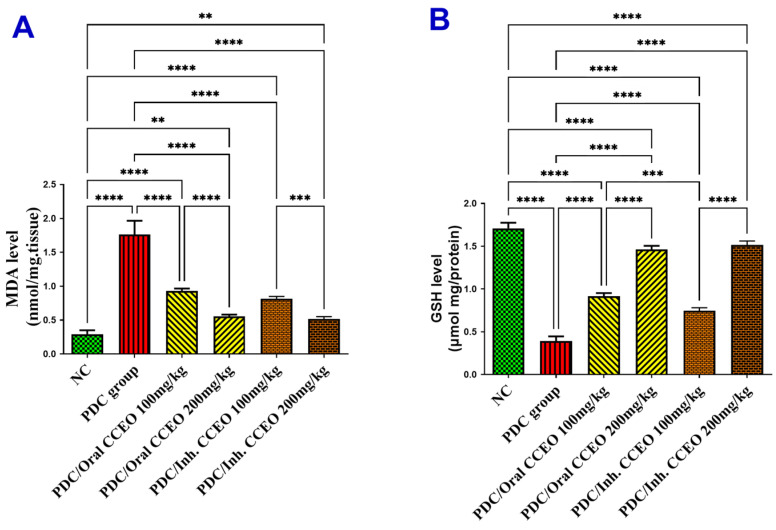
Effect of CCEO on (**A**) MDA and (**B**) GSH levels in the lung tissue homogenate. Data are expressed as mean ± SD where n = 6. Statistical analysis was performed using the one-way analysis of variance (ANOVA) followed by the Tukey’s multiple comparison test. ** *p* ≤ 0.01, *** *p* ≤ 0.001, **** *p* ≤ 0.0001. where, NC is the negative control group.

PDC-induced lung damage produced oxidative stress via a significant elevation of MDA and depletion of GSH lung contents by 507% and 77%, respectively, as compared with the data of the control group. The administration of both doses of CCEO by oral route led to a significant decrease in MDA lung content by 286% and 414%, respectively, restoring GSH enzyme activity by 31% and 63% in comparison with data of PDC animals. The administration of both doses of CCEO by inhalation route led to a significant restoration of the antioxidant state of lungs subjected to in-PDC as revealed by the decline in MDA lung content by 324% and 428%, respectively, restoring GSH enzyme activity by 21% and 66% in comparison with data of PDC animals (Figure 2).

##### Assessment of TNF-α and TGF-β Lung Contents

**Figure 3:**
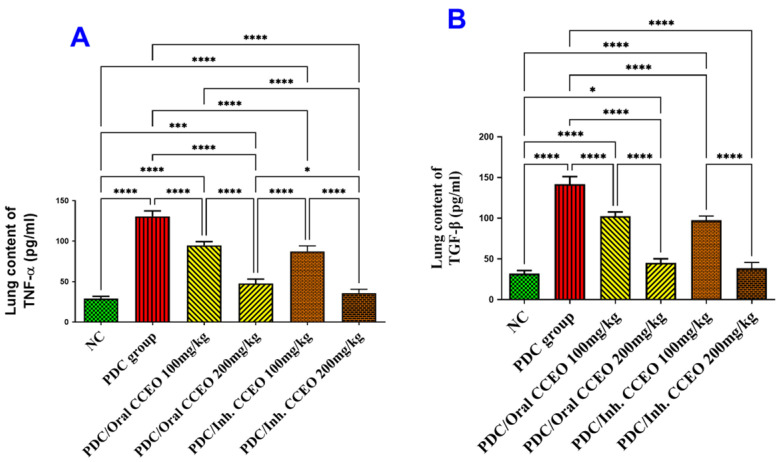
Effect of CCEO on (**A**) TNF-α and (**B**) TGF-β in the lung. Data are expressed as mean ± SD where n = 6. Statistical analysis was performed using the one-way analysis of variance (ANOVA) followed by Tukey’s multiple comparison test. * *p* ≤ 0.05, *** *p* ≤ 0.001, **** *p* ≤ 0.0001. Where, NC is the negative control group.

PDC-induced lung damage produced inflammation via a significant elevation of TNF-α and TGF-β lung contents by 348% and 345%, respectively, as compared with the data of the control group. The administration of both doses of CCEO by oral route led to a significant decrease in TNF-α lung content by 123% and 284% respectively, and TGF-β by 123% and 304% in comparison with data of PDC animals. The inhalation of 100 mg CCEO led to a significant decrease in TNF-α lung content by 148% respectively, and TGF-β by 140% in comparison with data of PDC animals. The TNF-α and TGF-β lung contents remained at the normal level of the control group, after treatment with 200 mg/kg by either the oral or inhalation CCEO routes. But the marked decrease in both TNF-α and TGF-β lung contents were achieved by inhalation of 200 mg/kg CCEO, where we recorded declines of 325% and 324% in comparison with data of PDC animals (Figure 3A,B).

##### Assessment of PI3K Lung Content

**Figure 4:**
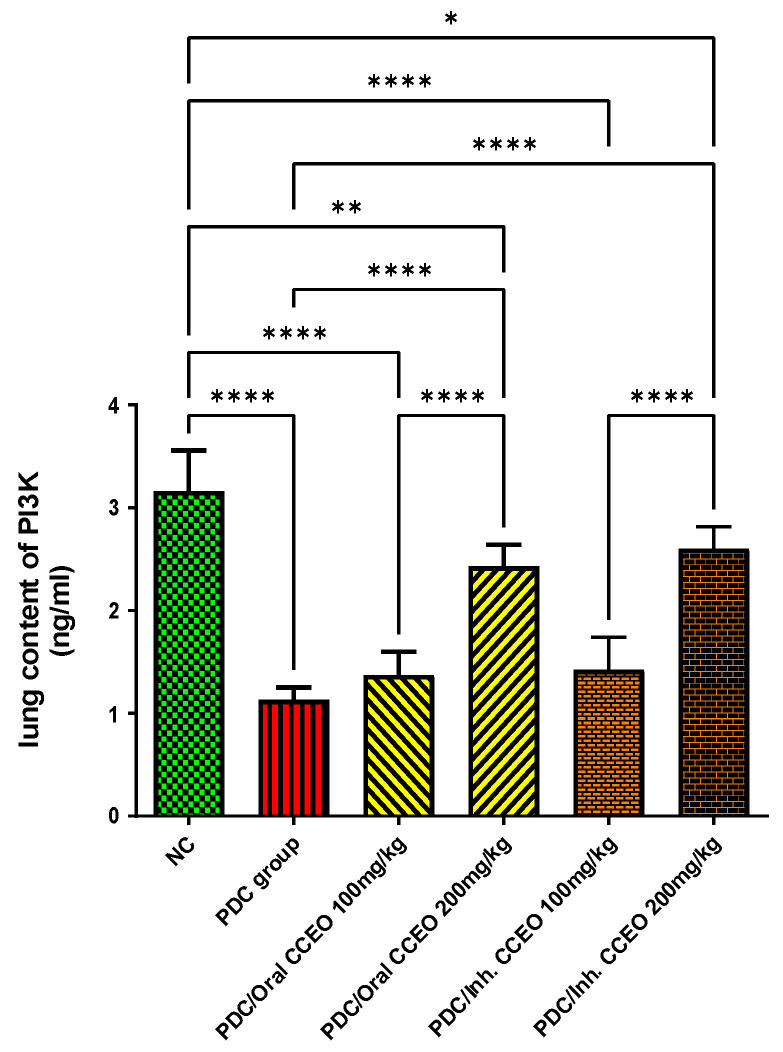
Effect of CCEO on PI3K in the lung. Data are expressed as mean ± SD where n = 6. Statistical analysis was performed using the one-way analysis of variance (ANOVA) followed by the Tukey’s multiple comparison test. * *p* ≤ 0.05, ** *p* ≤ 0.01, **** *p* ≤ 0.0001. Where, NC is the negative control group.

PDC-induced lung damage decreased PI3K lung contents by 180%, as compared with the data of the control group. The oral administration of both doses of CCEO led to a significant increase in brain contents of PI3K by 57% and 153%, respectively, in comparison with data of PDC animals. The inhalation of both doses of CCEO led to a significant increase in lung contents of PI3K by 61% and 158%, respectively, in comparison with data of PDC-treated animals. The lung contents of PI3K remained at the normal level of the control group, after treatment with the high dose of CCEO (Figure 4).

##### AKT Gene Expression Assessment

**Figure 5:**
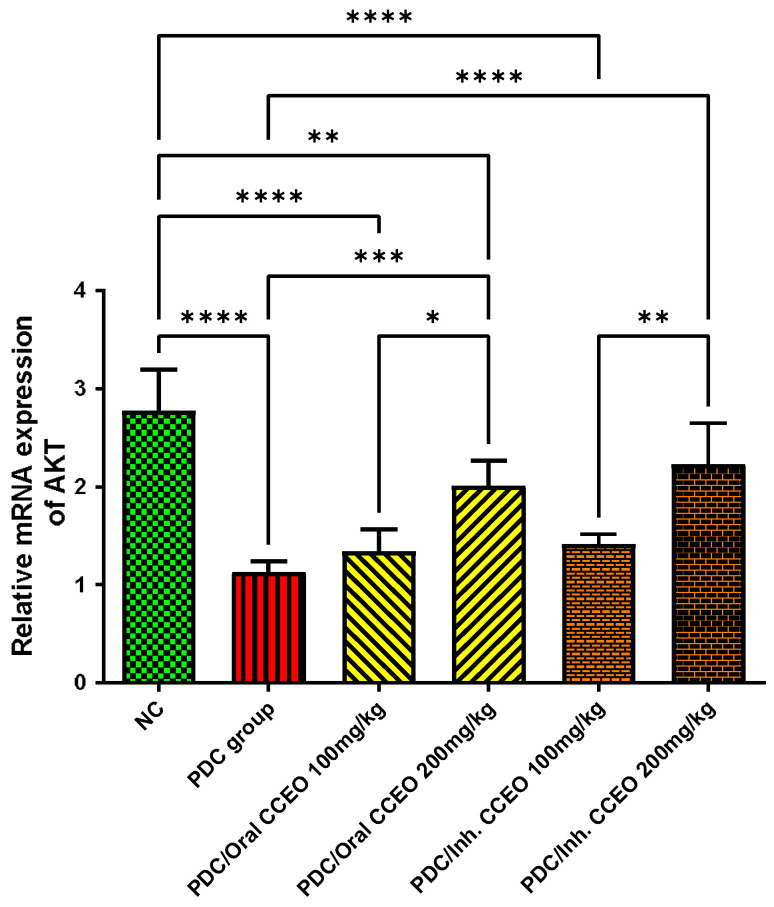
Effect of CCEO on AKT gene expression. Data are expressed as mean ± SD where n = 6. Statistical analysis was performed using the one-way analysis of variance (ANOVA) followed by Tukey’s multiple comparison test. * *p* ≤ 0.05, ** *p* ≤ 0.01, *** *p* ≤ 0.001, **** *p* ≤ 0.0001. Where, NC is the negative control group.

PDC reduced AKT gene expression by 145% as compared with the data of the control group. The oral administration of both doses of CCEO upregulated gene expression of AKT in the lungs by 54% and 123%, respectively, in comparison with data of PDC-treated animals. The inhalation of CCEO in two varied doses upregulated gene expression of AKT in the lungs by 67% and 127%, respectively, in comparison with data of PDC-treated animals. The gene expression of AKT remained at the normal level of the control group, after treatment with the high dose of CCEO either by oral or inhalation administration (Figure 5).

##### Histopathological and Immunohistochemical Results

**Figure 6:**
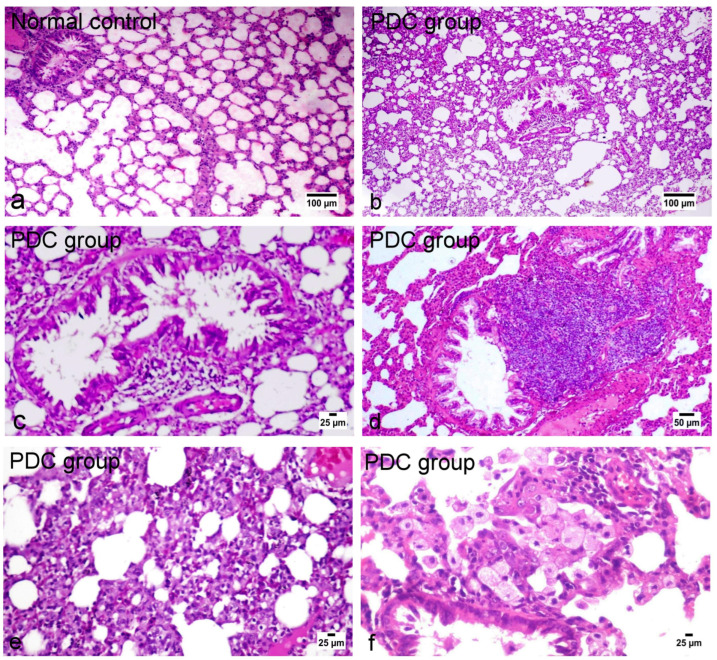
H&E-stained photomicrographs of the lung tissue of normal control and PDC-treated rats. (**a**) Normal histological appearance of both bronchioles and alveoli; (**b**–**f**) marked histological alterations of both bronchioles and alveoli.

**Figure 7:**
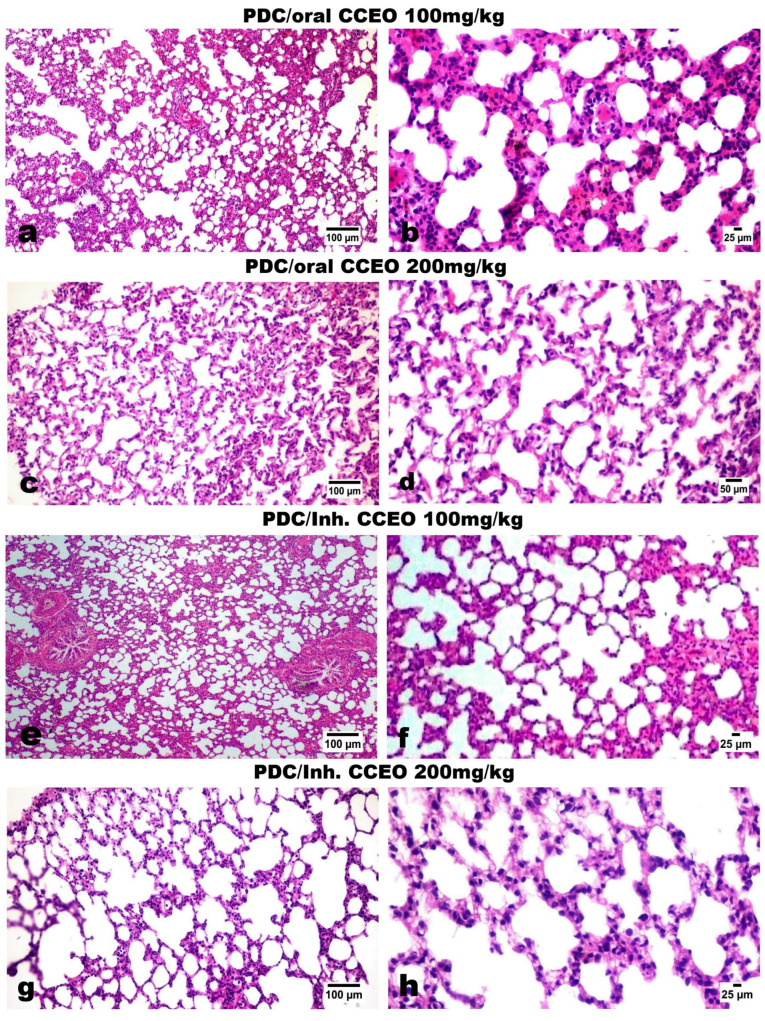
H&E-stained photomicrographs of the lung tissue of oral and inhalation-treated groups with CCEO. (**a**,**b**) Bronchi and alveoli treated with the low dose of CCEO orally (100 mg/kg bw). (**c**,**d**) Bronchi and alveoli treated with the high dose of CCEO orally (200 mg/kg bw). (**e**,**f**) The low dose of CCEO inhalation-treated groups (100 mg/kg). (**g**,**h**) The high dose of CCEO inhalation-treated groups (200 mg/kg).

**Figure 8:**
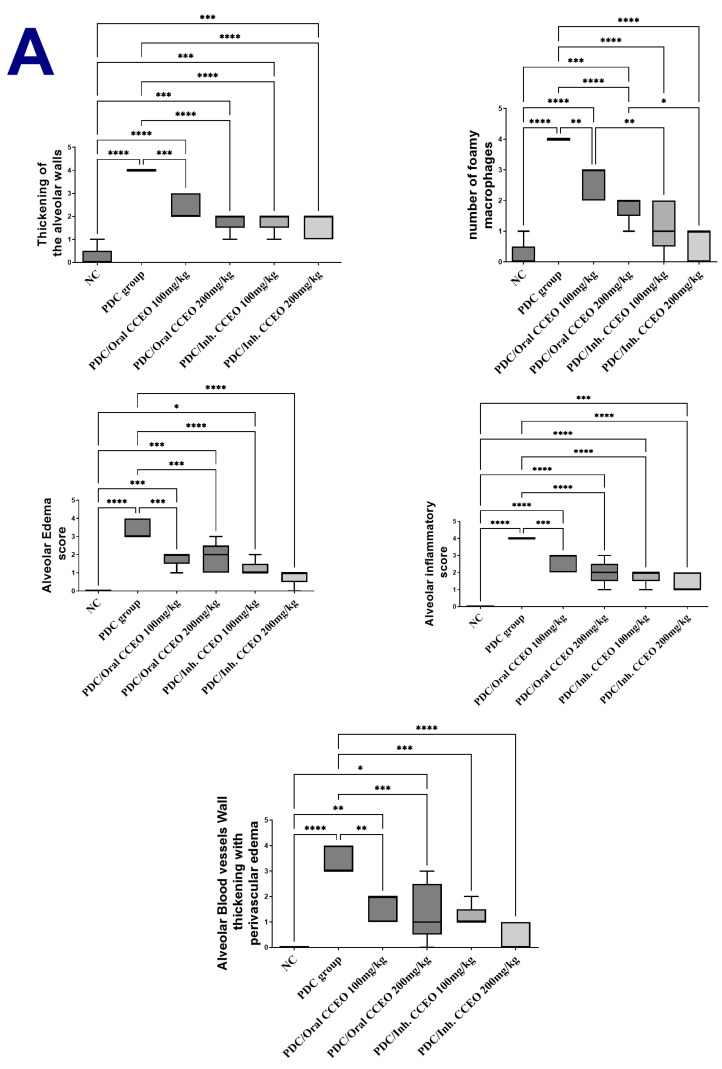

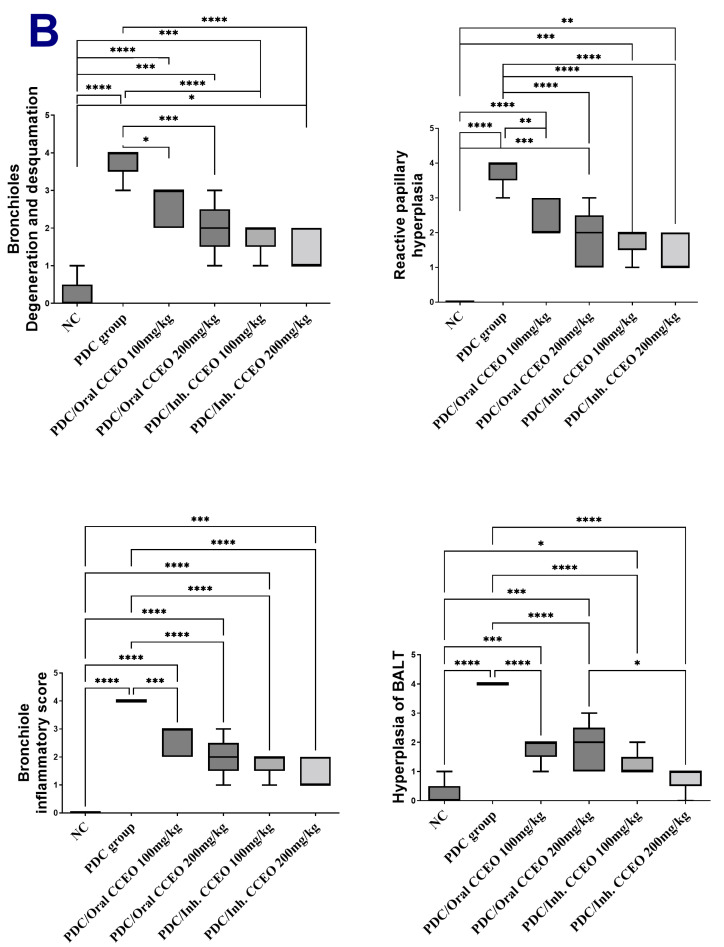
The scores of (**A**) alveolar tissue and (**B**) bronchial tissue reaction for intranasal administration of saline and PDC in different treated groups compared to the PDC model group. Scoring data are presented as median (max–min) using the Kruskal–Wallis test followed by the Mann–Whitney U test. Data are expressed as nonparametrically (median ± IQR) where n = 6. Statistical analysis was performed using the one-way analysis of variance (ANOVA) followed by Tukey’s multiple comparison test. * *p* ≤ 0.05, ** *p* ≤ 0.01, *** *p* ≤ 0.001, **** *p* ≤ 0.0001. Where, NC is the negative control group.

The lung tissue of normal control rats showed normal histological appearance of both bronchioles and alveoli (Figure 6a). On the other hand, the lungs of PDC-treated rats showed marked histological alterations as thickening of the interalveolar septa and bronchial epithelial reaction (Figure 6b). The bronchioles showed focal to diffuse papillary hyperplasia with some desquamated epithelial cells admixed sometimes with mucous exudate in the bronchiolar lumens (Figure 6c) with peri-bronchial inflammatory cell infiltration. Hyperplasia of the peri-bronchial lymphoid tissue was an evident finding (Figure 6d). Vascular reaction was evident characterized by thickening of the blood vessel walls and perivascular edema. The alveolar walls revealed marked diffuse thickening with intense mononuclear inflammatory cell infiltration and mild fibroplasia (Figure 6e) with intra-alveolar luminal aggregation of a large number of foamy macrophages (Figure 6f). Regarding the treated groups with limonene, the examination of various lung tissues revealed that the inhalation route for limonene administration had a more dose-related curative effect than the oral route of administration. Concerning the later treated (oral route) groups, their lungs showed mild to moderate degrees of relief of alveolar wall thickening, with decreased number of inflammatory cells infiltrating the alveolar walls and mild hyperplasia of the bronchial epithelium (Figure 7a–d) as well as conspicuous retraction of the peri-bronchial tissue hyperplasia, particularly in the high-dose treatment group. The inhalation route treatment groups revealed marked dose-related retraction of alveolar wall thickening (Figure 7e–h), at which the higher dose group showed near-normal thickness of the alveolar walls with very mild proliferation of the bronchial epithelial linings. Scarce vascular reaction was noticeable as slight perivascular edema, and no obvious peri-bronchial inflammatory cell infiltration or hyperplasia of the peri-bronchial lymphoid tissue were observed. The scorings of the bronchial and alveolar histological changes are presented in Figure 8A,B, in normal control and various experimental groups.

**Figure 9:**
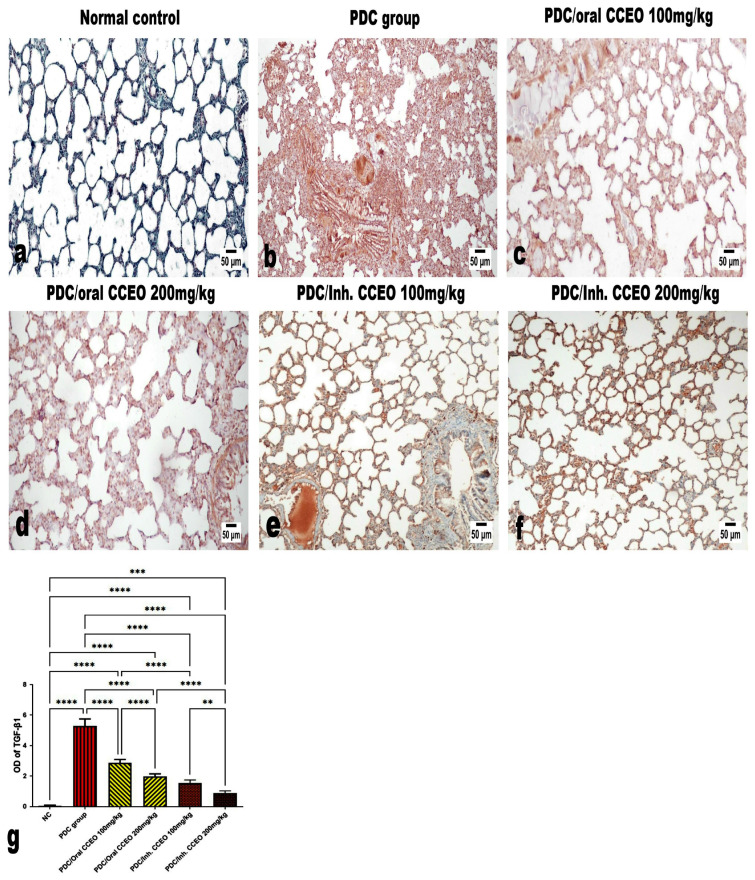
Immunohistochemistry of lung tissue showing the immune expression of TGF-β1. (**a**,**b**) The PDC-administrated group. (**c**) The low dose of CCEO orally treated groups (100 mg/kg). (**d**) The high dose of CCEO orally treated groups (200 mg/kg). (**e**) The low dose of CCEO inhalation-treated groups (100 mg/kg). (**f**) The high dose of CCEO inhalation-treated groups (200 mg/kg). (**g**) Quantitative analysis of the positive brown color intensity of TGF-β1 expression performed using image analysis software. ** *p* ≤ 0.01, *** *p* ≤ 0.001, **** *p* ≤ 0.0001. Where, NC is the negative control group.

Regarding the immune expression of TGF-β1 (Figure 9a–f), the PDC-administrated group showed an intensive diffuse expression of TGF-β1 in relation to the control and the limonene-treated groups. The least significant immune expression was observed in the inhalation-treated groups, particularly those that received the higher dose limonene. The quantitative analysis of the positive brown color intensity of TGF-β1 expression was performed using image analysis software (Figure 9g).

## 4. Discussion

In this study, the essential oils extracted from *Citrus clementine* were found to be rich in limonene, with similar findings reported by previous studies [8,18,19,20]. These studies demonstrated that limonene content ranged from 86% to 93% in clementine peel essential oils. On the other hand, the second abundant metabolite, which represented 3.94% in our work, was reported for its potential anti-inflammatory activity and ranged from 1 to 9% in CCEO content [8,19,20,21,22]. Generally, our results were similar to [23], in which the main constituents of CCEO, obtained from Vietnam, were limonene, myrcene, and α-pinene

Moreover, the limonene-rich essential oils showed antioxidant activity by increasing the level of GSH and decreasing the level of MDA. Various studies have explored the antioxidant activity of *Citrus* and its potential benefits in ALI. These studies have shown promising results, indicating the protective effects of *Citrus* against oxidative stress-induced lung injury [24,25,26]. However, it should be noted that some studies have suggested that antioxidants may not have a significant impact on severe organ damage and could potentially worsen organ injury and decrease the survival rate, particularly when combined with high-risk diseases like heart failure [27,28,29]. The antioxidant activity of *Citrus clementine* can be attributed to its composition of essential oils, particularly monoterpenes such as limonene and linalool. These compounds have strong antioxidant properties and have been reported to exhibit anti-inflammatory effects. They can help reduce oxidative stress and inflammation in lung tissue, potentially alleviating ALI [30].

Limonene exhibits antioxidant activity through several mechanisms. Firstly, it functions as a free radical scavenger, effectively neutralizing reactive oxygen species (ROS) and preventing oxidative damage. Limonene’s scavenging ability helps protect cells and tissues from oxidative stress [31]. Secondly, limonene enhances the activity of endogenous antioxidant enzymes, including superoxide dismutase (SOD), catalase (CAT), and glutathione peroxidase (GPx). These enzymes play a crucial role in the cellular defense against oxidative stress by converting harmful ROS into less reactive molecules. Moreover, limonene can modulate the expression of genes involved in antioxidant defense pathways. It has been shown to upregulate the expression of genes encoding antioxidant enzymes, such as SOD and GPx, thereby enhancing the cellular antioxidant capacity [32].

Limonene possesses anti-inflammatory effects in addition to its antioxidant properties. Chronic inflammation can lead to increased oxidative damage, and by reducing inflammation, limonene indirectly contributes to antioxidant activity by minimizing the production of pro-inflammatory mediators that can trigger oxidative stress. It regulates various inflammatory signaling pathways, including NF-κB, MAPKs, and cytokines. Limonene inhibits NF-κB activation, a transcription factor involved in pro-inflammatory gene expression, thereby preventing the release of inflammatory mediators [33]. It also suppresses the production of cytokines involved in the inflammatory response, such as TNF-α, TGFβ, IL-6, and IL-1β, thereby reducing inflammation and oxidative stress [34]. The antioxidant activity of limonene, including its ability to scavenge free radicals, enhance antioxidant enzyme activity, and modulate gene expression related to antioxidant defense pathways, makes it a valuable compound for potential therapeutic applications. Inhaling citrus essential oils high in terpenes, such as limonene, can enhance the production of glutathione (GSH) and protect against oxidative damage to fats in the brain. This helps prevent DNA damage and cell death by eliminating harmful free radicals (ROSs) through their antioxidant properties. Inhalation of these essential oils also increases the levels of antioxidant enzymes like SOD, glutathione peroxidase, and CAT, which play a role in the immune system. Moreover, the terpenes found in citrus essential oils have been shown to alleviate inflammation symptoms by reducing the release of inflammatory molecules like NF-κB, IL-1β and TNF-α [30].

One of the most significant findings in this study is that CCEO activated the PI3K/AKT pathway. This study supports evidence from previous observations [35] which reported the impact of the activation of the phosphoinositide 3-kinase (PI3K)/Akt signaling pathway as a protective mechanism against pulmonary ischemia-reperfusion (IR) injury. Moreover, other studies revealed the importance of the PI3K/AKT pathway activation to reduce the damage of ALI [36,37]. Although there is limited research on the specific relationship between potassium dichromate (PDC) and the PI3K/AKT pathway, studies on other lung injury models and toxicants suggest potential interactions. For example, paraquat-induced lung injury [38] and silica-induced lung injury [39] have implicated the PI3K/AKT pathway in the pathogenesis and regulation of inflammatory responses. Moreover, inhibition of the PI3K/AKT pathway has been found to exacerbate lung injury and inflammation.

The PI3K/AKT pathway is one of the signaling pathways associated with oxidative stress and lung injury. It plays a crucial role in cell survival, proliferation, metabolism, and apoptosis. Activation of AKT can directly modulate the cellular response to oxidative stress and protect against oxidative damage. In the context of lung injury, the PI3K/AKT pathway has been implicated in regulating oxidative stress-induced lung damage and inflammation [40]. Activation of the PI3K/AKT pathway in lung cells can occur through various mechanisms, including growth factor receptor activation, cytokine signaling, and oxidative stress itself. Once activated, AKT exerts its protective effects by phosphorylating and activating various downstream targets involved in antioxidant defense, inflammation regulation, and cell survival [41,42]. The pathway also promotes the nuclear translocation and activation of Nrf2, leading to the induction of genes encoding antioxidant enzymes and enhancing the cellular antioxidant capacity [43]. Studies have shown that modulating the PI3K/AKT pathway can attenuate oxidative stress, reduce lung tissue damage, and improve lung function in various lung injury models [44,45].

The results of this study revealed both oral and inhalation use of limonene ameliorates the destructive effect of PDC-induced ALI. However, the inhalation route is preferred for administering limonene to maximize the effectiveness of treatment and minimize the occurrence of adverse reactions [46]. This route of administration ensures a high concentration of limonene at the targeted location while minimizing its absorption in other organs, in addition to the rapid onset compared to the oral route [47]. The lungs possess favorable characteristics for drug absorption, including a large surface area, a thin epithelium, and a high rate of blood perfusion. These factors enable rapid systemic circulation of drugs, making inhalation the optimal choice for treating respiratory disorders [48].

## 5. Conclusions

The findings of this study provide initial evidence indicating that limonene-rich essential oils extracted from *Citrus clementine* peels offer beneficial effects in mitigating acute ALI caused by PDC. These effects are primarily attributed to the antioxidant and anti-inflammatory properties of limonene-rich essential oils as well as activating PI3K/AKT signaling pathways. Furthermore, limonene demonstrated a significant reduction in histological damage modulating structural changes in lung tissue and biochemical changes within the respiratory system induced by PDC. Hence, CCEO has a promising protective effect for diminishing pulmonary injury through both oral and inhalation delivery systems.

## Figures and Tables

**Table 1:** Essential oil constituents of *Citrus clementine* analyzed by GC/MS.

Peak	Retention Index (RI)		Area%	Identified Compounds	Chemical Class
	Reported	Literature			
1	928	932	2.48	α-Pinene	Monoterpene
2	959	969	1.77	Sabinene	Monoterpene
3	985	990	3.94	*β*-Myrcene	Monoterpene
4	1019	1024	88.84	D-Limonene	Monoterpene
5	1094	1095	0.86	Linalool	Oxygenated monoterpene
6	1434	1432	0.6	*β*-Copaene	Sesquiterpene
7	1532	1529	0.64	*β*-Cadinene	Sesquiterpene
8	1699	1997	0.87	*β*-Sinensal	Oxygenated Sesquiterpene

## Data Availability

No new data were created or analyzed in this study. Data sharing is not applicable to this article.
